# Effects of Abrasive Flow Finishing on the Surface Quality, Frictional Resistance, and Biological Performance of Brackets

**DOI:** 10.1016/j.identj.2026.109721

**Published:** 2026-07-03

**Authors:** Fan Yang, Chen Zhou, Zichun Huang, Meihan Chen, Yang Cao, Lili Chen, Weicai Wang

**Affiliations:** Hospital of Stomatology, Guanghua School of Stomatology, Guangdong Provincial Key Laboratory of Stomatology, Sun Yat-sen University, Guangzhou, Guangdong, China

**Keywords:** Orthodontic brackets, Abrasive flow finishing, Surface roughness, Frictional resistance

## Abstract

**Objectives:**

This study aims to investigate the effects of abrasive flow finishing (AFF) on the surface quality, frictional resistance, and biological performance of orthodontic brackets.

**Methods:**

Stainless-steel brackets were randomly allocated into blank, control, and AFF groups. The blank group was left untreated. The control group underwent conventional magnetic polishing, while the AFF group was treated with AFF. Surface morphology was observed using scanning electron microscopy. Surface roughness at the micro- and nanoscale was investigated *via* 3D optical profilometry and atomic force microscopy, respectively. The arithmetic average roughness (Ra) and root mean square roughness (Rq) were analysed. The changes in slot dimensions were evaluated. Frictional resistance was determined using a universal testing machine. Biological performance was evaluated *via* bacterial adhesion and *in vitro* calculus formation assays.

**Results:**

The AFF group presented a mirror-like finish. Scanning electron microscopy micrographs exhibited the smooth and uniform surface in outer and slot areas in the AFF group, while the control group showed obvious defects and pits within slots. 3D optical profilometry and atomic force microscopy demonstrated the smooth and regular surface topography in the AFF group. Quantitative analysis showed that Ra and Rq values of the slot area in the AFF group were significantly lower than those of blank and control groups. There were no significant changes in slot dimensions following AFF treatment. The kinetic friction force was significantly reduced in the AFF group. Furthermore, AFF treatment inhibited bacterial adhesion and calculus-like mineral deposition on brackets.

**Conclusion:**

AFF treatment effectively reduced the surface roughness of orthodontic brackets, especially within slot area, outperforming conventional magnetic polishing. This improvement further minimized frictional resistance and inhibited bacterial adhesion and calculus-like mineral deposition.

**Clinical relevance:**

This study demonstrates that AFF is a superior post-processing technique for orthodontic brackets.

## Introduction

In fixed orthodontic treatment, excessive friction force between archwires and brackets impedes sliding mechanics and is associated with root resorption and anchorage loss.^1^^,^^2^ Consequently, minimizing this friction is the key to enhancing treatment efficiency and efficacy.

Brackets are fundamental components of orthodontic appliances. While the surface roughness of brackets substantially impacts frictional resistance,^3^^,^^4^ current manufacturing techniques like casting, metal injection moulding, and computer-aided design and manufacturing inevitably introduce surface defects, burrs and asperities.^5^, ^6^, ^7^, ^8^, ^9^, ^10^ Such irregularities not only exacerbate frictional resistance, but also elevate the risk of bacterial adhesion that could induce gingival inflammation, enamel decalcification and dental calculus formation.^11^^,^^12^ Therefore, effective post-fabrication polishing is an essential prerequisite for the clinical application of orthodontic brackets.

In order to reduce the surface roughness, various post-processing polishing techniques have been developed, such as mechanical buffing, electropolishing, and magnetic abrasive finishing.^13^^,^^14^ Nevertheless, the complex geometries of brackets with intricate structures render the finishing process challenging. Although conventional finishing techniques could access the external surface of brackets, they often fail to polish the intricate structures like slots effectively.^4^^,^^15^ Thus, an innovative finishing approach capable of achieving high-level polishing across complex geometries is warranted.

Abrasive flow finishing (AFF) is an advanced finishing technology that is widely applied in aerospace, electronics, and automotive manufacturing.^16^ In this process, a semisolid abrasive medium consisting of viscoelastic polymers and abrasive particles is extruded across the target surface under hydraulic pressure to produce controlled microcutting and smoothing. Leveraging the fluidic nature of the abrasive medium, AFF is able to polish complex internal structures that are inaccessible to conventional techniques.^16^^,^^17^ Despite these potential advantages, the application of AFF for the surface finishing of orthodontic brackets remains unexplored.

The objective of this study was to comprehensively evaluate the application of AFF as a post-fabrication finishing strategy for orthodontic brackets. Surface morphology, roughness, changes in slot dimensions, and frictional resistance were systematically analysed following AFF treatment. Furthermore, the effects of AFF on bacterial adhesion and calculus formation were investigated. This work aims to present a novel polishing strategy to improve the mechanical and biological performance of orthodontic brackets.

## Materials and methods

### Study design

Stainless-steel brackets (YL, XIHUBIOM) with a slot size of 0.020 × 0.028 inches were used in this study. These brackets were fabricated using the metal injection moulding technique and possessed complex geometries. The brackets were randomly allocated into the blank, control, and AFF groups (*n* = 3). The regions of interest included the outer surface and the slot area (Figure S1).

### Polishing procedures

The blank group received no surface treatment. Brackets in the control group were processed by conventional magnetic polishing technique. The frequency control magnetic polishing grinder (HYT, 360D) was utilized according to manufacturer’s instructions. The abrasive medium consisted of magnetic steel needles (0.3 mm in diameter, 5 mm in length) with LGL-5 polishing fluid. The finishing process was performed at a rotational speed of 1800 rpm for 1 hour.

Brackets in the AFF group were finished using the abrasive flow polishing machine (SMKS, B700×2) (Figure S2A-C) according to manufacturer’s instructions. The brackets were fixed within a customized tooling fixture (Figure S2B). The abrasive medium was composed of garnet (35 wt%), silicon dioxide (28 wt%), rubber (22 wt%), and mineral oil (15 wt%) (Figure S2C). The working load was 100 kg. The polishing process consisted of 30 cycles over 20 minutes.

### Scanning electron microscope observation

The morphology and surface structure of brackets were examined using scanning electron microscopy (SEM) (Apreo2, Thermo Fisher) at the accelerating voltage of 10 kV.

### 3D optical profiler evaluation

The roughness of brackets at microscale was analysed using a 3D optical surface profiler (Contour GT-K 3D, Bruker) in the vertical shift interference mode. The arithmetic average roughness (Ra) and root mean square roughness (Rq) values for the outer surface and the slot area were evaluated. Ra indicates the average absolute deviation of roughness from the mean line, while Rq indicates the root mean square of height deviations.

### Atomic force microscope analysis

The surface roughness at nanoscale was investigated *via* atomic force microscopy (AFM) (Dimension Icon, Bruker). The micrographs of the outer surface and slot area were obtained with the scan area of 20 × 20 μm. The Ra and Rq values were calculated from the topographical data.

### Slot dimensions evaluation

To investigate the surface wear following polishing, the slot depth was evaluated using a 3D optical surface profiler (Contour GT-K 3D, Bruker). The brackets were placed horizontally on the stage. Then, the vertical distance between the slot bottom and the slot top was measured (Figure S3A). The slot height was evaluated using a microscope (BX53, Olympus). The brackets were placed vertically on the table. Then, the micrographs of the slot were captured. The shortest distance between the upper surface and lower surface was measured (Figure S3B).

### Frictional resistance analysis

The frictional resistance was investigated using a universal testing machine (E3000, Instron). Prior to testing, stainless-steel archwires (0.018 × 0.025 inches) and brackets were cleaned with 75% ethanol. Each bracket was bonded to a plastic plate (10 × 10 × 1 mm) by cyanoacrylate adhesive and mounted on the lower fixture of tension-loading cell. The archwire was ligated to the bracket using an elastic ligature (Ormco). To ensure the consistency of the normal force, all ligations were performed immediately prior to friction analysis. To reduce the operator-dependent variation, all ligations were made by the same investigator. Each elastic ligature was used only once. The upper end of the archwire was fixed in the upper fixture of tension-loading cell with zero angulation and torque. The archwire was drawn through the bracket at the speed of 1 mm/min with the 100 N load cell for 5 minutes, while the kinetic friction force was recorded. The average friction force between the second and fourth millimetres of displacement was evaluated.

### Bacterial adhesion assay

Brackets were incubated with *Staphylococcus aureus* (*S. aureus*) (1 × 10^4^ CFU/mL) for 3 days at 37°C. Then, brackets were rinsed with PBS three times, followed by immobilization using glutaraldehyde at 4°C overnight. After serial dehydration, the bacteria on the surface of brackets were investigated under SEM (Crossbeam 550, Zeiss). To quantitatively assess bacterial adhesion, the bacterial coverage area in the SEM micrographs was analysed using ImageJ (NIH).

### *In vitro* calculus formation assay

*In vitro* calculus formation was simulated using a cyclic mineralization method as previously described.^18^^,^^19^ Initially, brackets were treated with artificial saliva (PH1843, Phygene) at 37°C for 6 hours to form the acquired pellicle. Afterwards, brackets were incubated with mineralizing solution containing NaHCO_3_ (0.84 g/L), MgCl_2_ (0.21 g/L), KH_2_PO_4_ (0.07 g/L), CaCl_2_ (0.28 g/L), and NaCl (4.92 g/L) at 37°C for 18 hours in a shaker (200 rpm). This cycle of the acquired pellicle formation and mineralization was repeated five times. Then, brackets were collected and rinsed with distilled water three times. Following serial dehydration, the calculus-like deposits on brackets were observed using SEM (Apreo2, Thermo Fisher). The elemental weight percentage of Ca, Mg, and P on the surface of brackets was evaluated using energy-dispersive spectroscopy.

### Statistical analysis

Data are expressed as mean ± standard deviation. Data analysis was performed using GraphPad (Prism 8). Statistical differences were analysed by one-way analysis of variance (ANOVA) followed by Tukey’s post hoc test. The *P* value < .05 was considered statistically significant. All experiments were replicated in three independent samples per group (*n* = 3).

## Results

### Surface morphology analysis

**Fig. 1:**
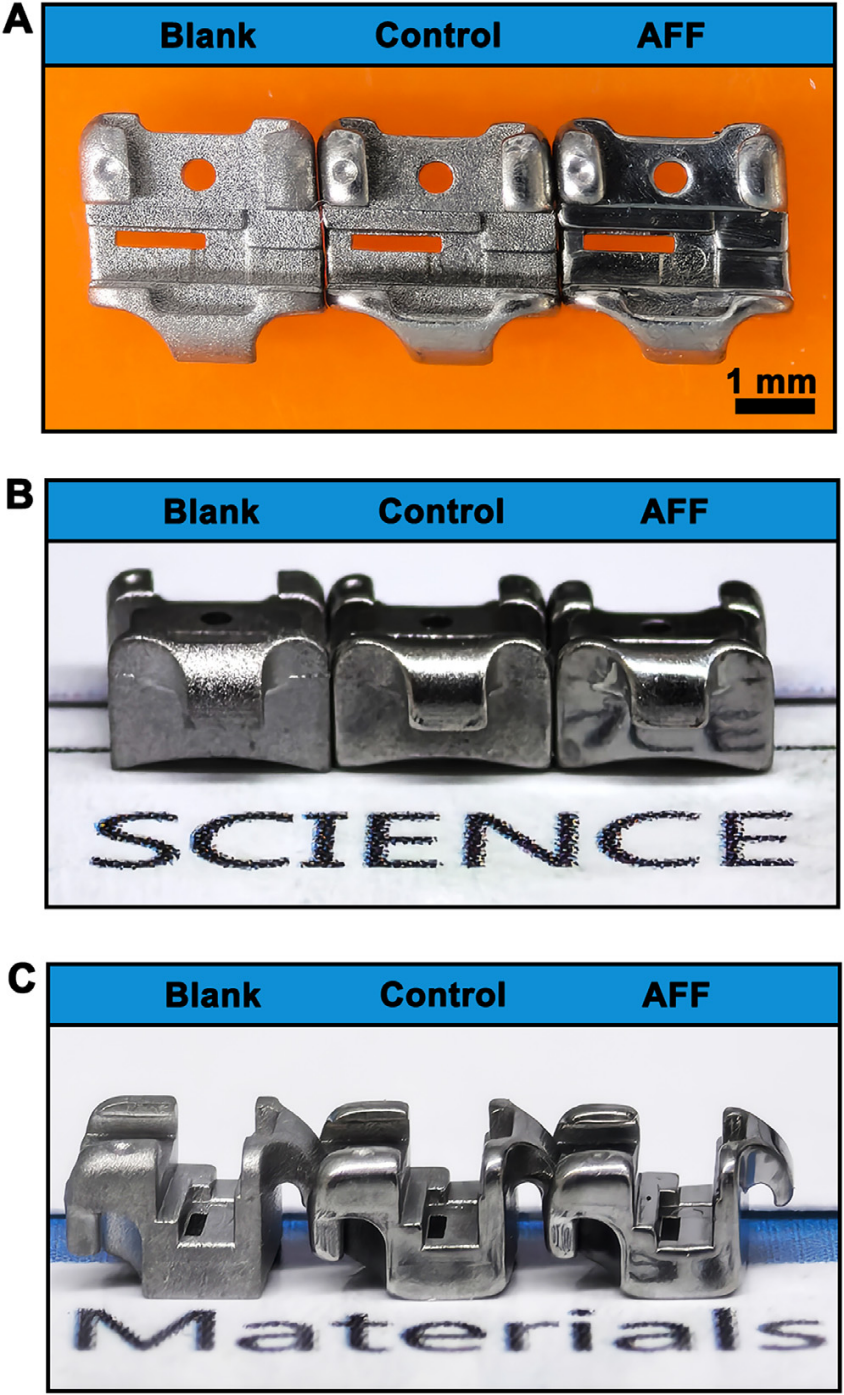
Photographs of brackets. (A) Top view. (B) Frontal view. (C) Lateral view.

The macroscopic appearance of the brackets in different groups is presented in Figure 1A. Following AFF treatment, the bracket surface exhibited a mirror-like finish capable of reflecting text (Figure 1B, C), indicating superior polishing effects.

**Fig. 2:**
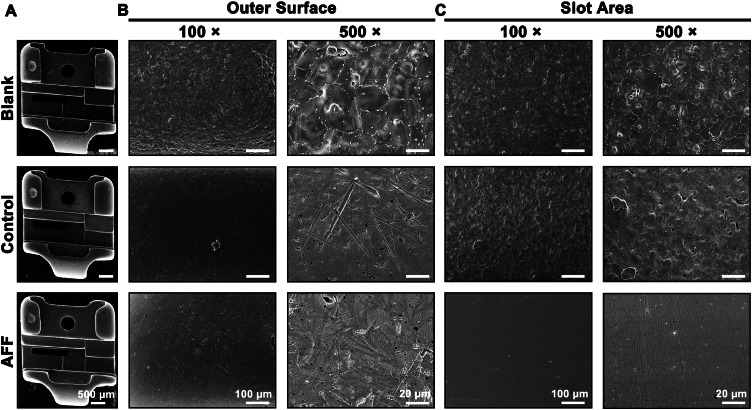
SEM micrographs of brackets. (A) Global morphology. (B) SEM micrographs of outer surface. (C) SEM micrographs of slot area.

Then, the surface morphology of the brackets was characterized using SEM (Figure 2A-C). Micrographs of the blank group showed evident grain boundaries and irregular protrusions on the surface (Figure 2B, C). Although the control group exhibited reduced irregularities on the outer surface (Figure 2B), there were obvious pits and defects on the surface of slot area (Figure 2C). In comparison, SEM micrographs showed smooth and regular topography on the external surface and slot area in the AFF group (Figure 2B, C).

### Roughness analysis

**Fig. 3:**
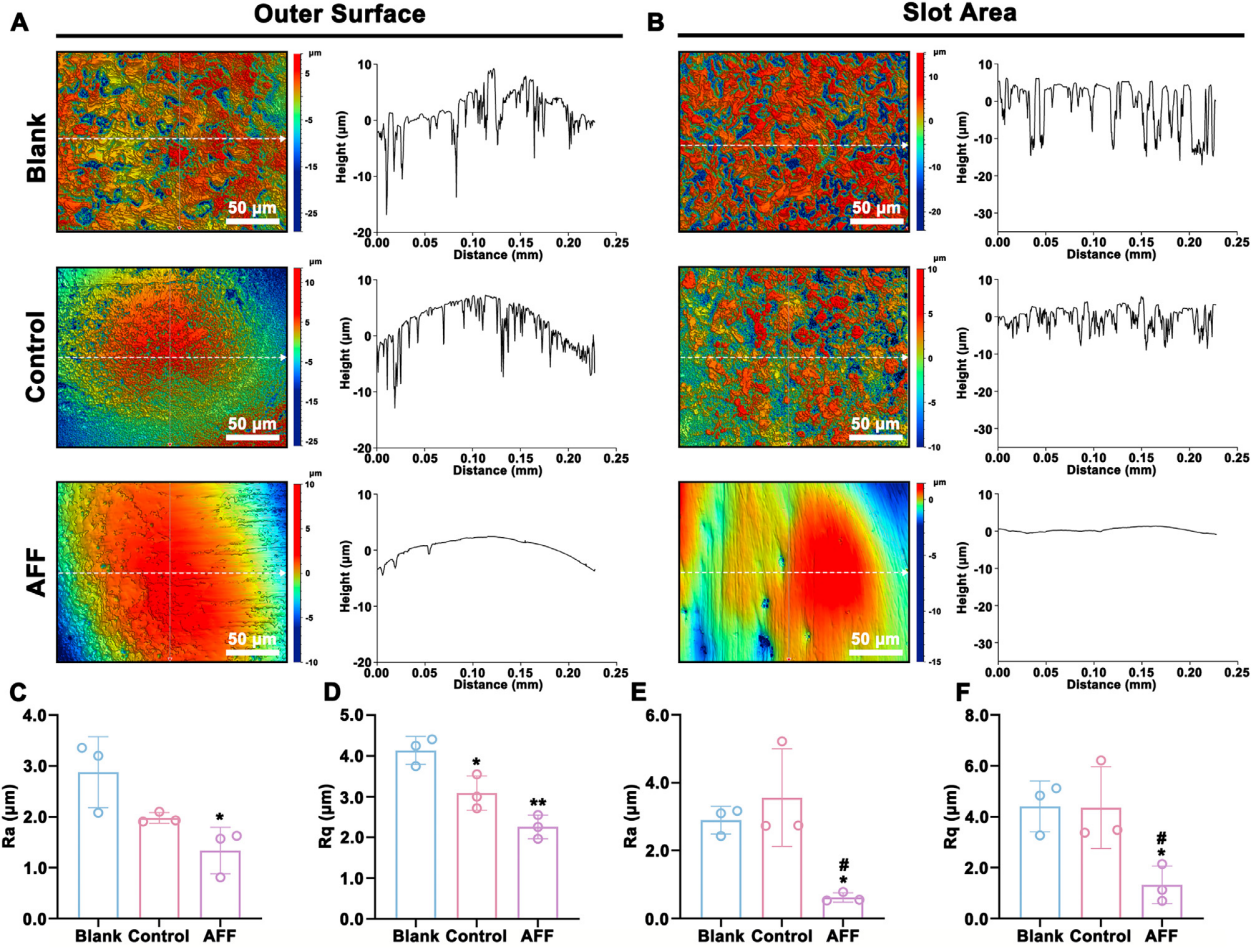
3D optical profiler analysis. (A) Representative 3D optical profiler micrographs of outer surface and corresponding height profiles. (B) Representative 3D optical profiler micrographs of slot area and corresponding height profiles. (C) Ra value of outer surface. (D) Rq value of outer surface. (E) Ra value of slot area. (F) Rq value of slot area. The white-dotted line indicates the evaluation cross-section. The colour bar indicates the height. *P < .05 compared with the blank group; **P < .01 compared with the blank group; ^#^*P* < .05 compared with the control group.

The surface roughness at the microscale was evaluated *via* 3D optical profilometry. The blank group exhibited highly irregular surface morphology characterized by sharp peaks and deep valleys (Figure 3A, B). Although the control group showed slightly reduced irregularity, it retained the rough surface in outer and slot areas (Figure 3A, B). In contrast, the AFF group demonstrated a dramatic improvement in surface quality, showing a smooth and uniform surface (Figure 3A, B). To quantitatively investigate the roughness, the Ra and Rq values were analysed (Figure 3C-F). The Ra and Rq values of the outer surface in the AFF group were significantly lower than those of the blank group, though no significant difference in the Ra and Rq values was observed between the control and AFF groups (Figure 3C, D). For the slot area, Ra and Rq values were significantly decreased in the AFF group compared to the other groups (Figure 3E, F).

**Fig. 4:**
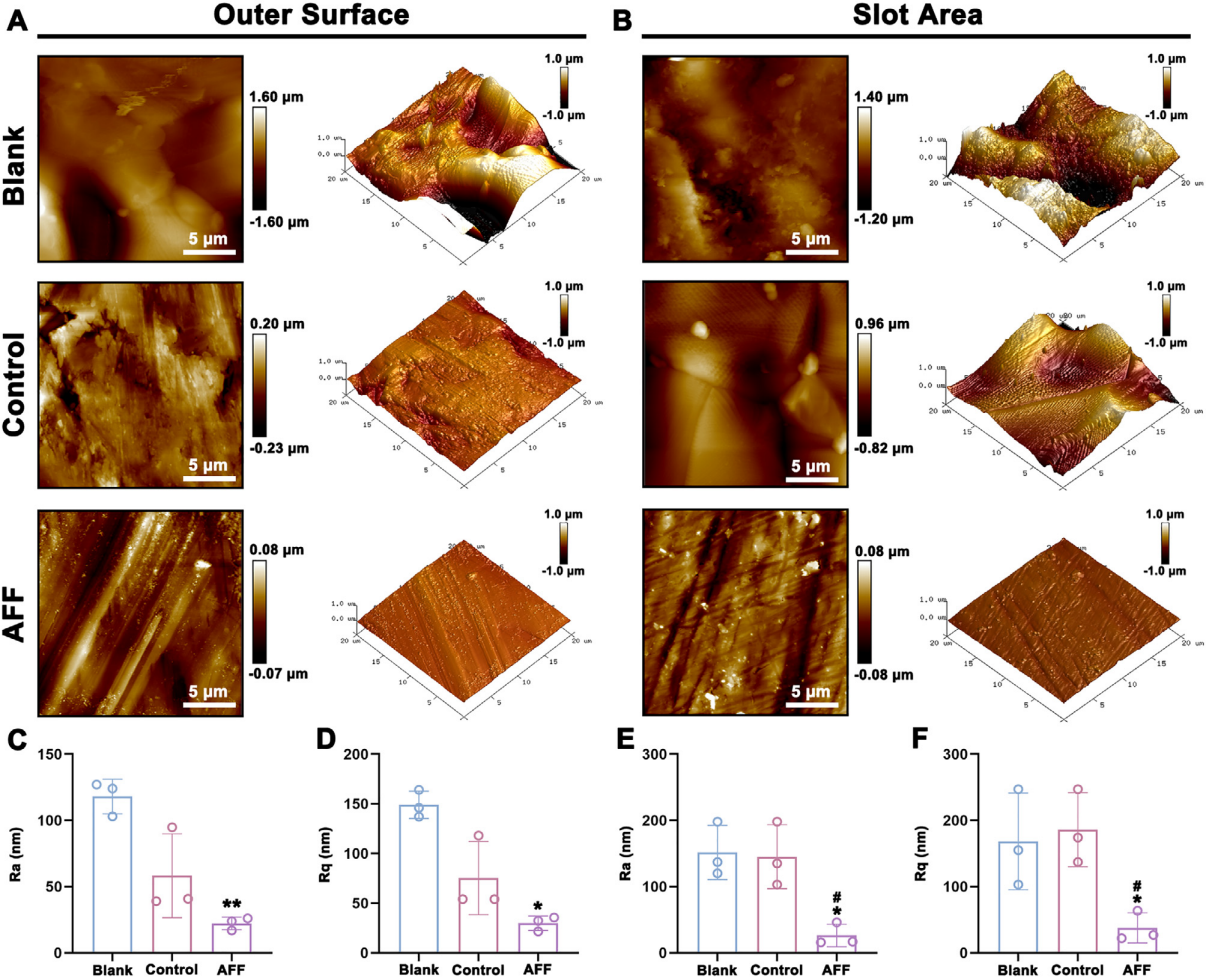
AFM investigation. (A) Representative AFM micrographs and 3D reconstruction of outer surface. (B) Representative AFM micrographs and 3D reconstruction of slot area. (C) Ra value of outer surface. (D) Rq value of outer surface. (E) Ra value of slot area. (F) Rq value of slot area. *P < .05 compared with the blank group; **P < .01 compared with the blank group; ^#^*P* < .05 compared with the control group.

Next, the surface roughness at the nanoscale was investigated *via* AFM. The 2D height maps and 3D reconstruction are shown in Figure 4A, B. The surface of brackets in the control group was smoother than that of the blank group, whereas irregularities characterized by pronounced peaks and valleys were still obvious within the slot area of the control group (Figure 4A, B), suggesting the limitations of conventional polishing in the intricate structures. Following AFF treatment, both the outer surface and slot area appeared more uniform and smoother than those of the other groups (Figure 4A, B). Quantitative analysis showed that there was no significant difference in Ra and Rq values between the blank and control groups (Figure 4C-F). Notably, AFF treatment resulted in obviously improved surface quality in the outer surface, achieving the Ra of 22 nm and the Rq of 30 nm (Figure 4C, D). In the slot area, the AFF group showed significantly lower Ra and Rq values compared to the other groups (Figure 4E, F).

### Slot dimensions evaluation

**Fig. 5:**
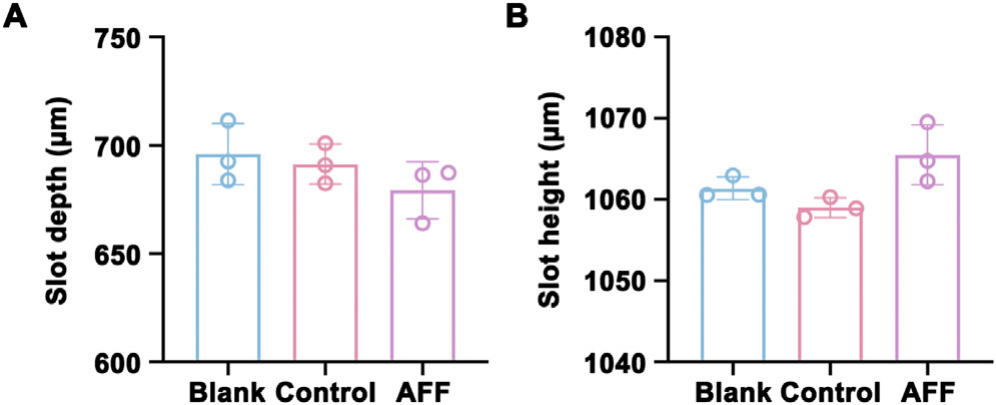
Evaluation of slot dimensions. (A) Evaluation of slot depth. (B) Evaluation of slot height.

The slot depth and height were evaluated using 3D profilometry and microscopy, respectively. As shown in Figure 5A, B, no significant differences were observed in either slot depth or height among the three groups.

### Frictional resistance evaluation

**Fig. 6:**
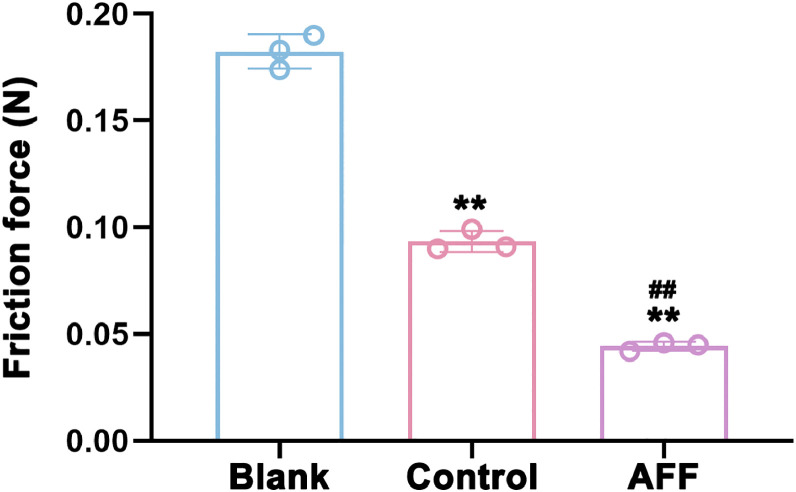
Analysis of kinetic friction force. **P < .01 compared with the blank group; ^##^*P* < .01 compared with the control group.

Following the confirmation of reduced surface roughness in the AFF group, the frictional resistance was evaluated. The results demonstrated that AFF treatment substantially reduced the frictional resistance between slots and stainless-steel archwires, resulting in significantly lower friction force than that of blank and control groups (Figure 6).

### Bacterial adhesion and calculus formation investigations

**Fig. 7:**
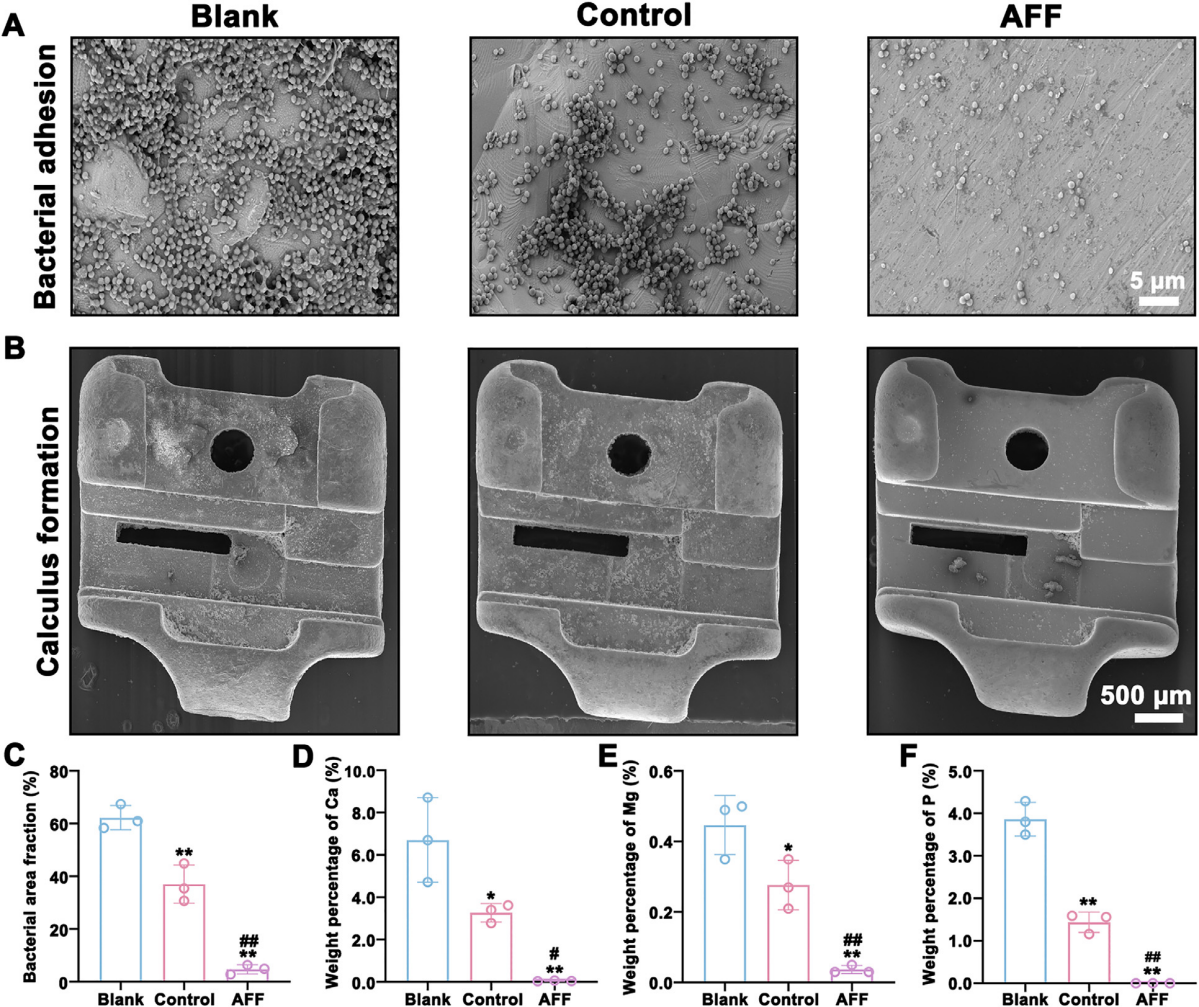
Biological performance evaluation. (A) SEM micrographs of brackets after coculturing with bacteria suspension. (B) SEM micrographs of brackets following mineralization treatment. (C) Bacterial area fraction. (D) Weight percentage of Ca on the bracket surface. (E) Weight percentage of Mg on the bracket surface. (F) Weight percentage of P on the bracket surface. *P < .05 compared with the blank group; **P < .01 compared with the blank group; ^#^*P* < .05 compared with the control group; ^##^*P* < .01 compared with the control group.

To investigate bacterial adhesion on the brackets, SEM micrographs were captured after coculturing the brackets with bacterial suspension. As shown in Figure 7A, extensive bacterial colonization was identified on the surfaces of brackets in both blank and control groups (Figure 7A, C). In contrast, only a few bacteria were attached to the bracket surface after AFF treatment (Figure 7A, C).

In calculus formation assay, the surfaces of blank and control groups were covered by calculus-like deposits after 5-cycle incubation (Figure 7B). However, the AFF group presented a much smoother surface with fewer calculus-like deposits (Figure 7B). The elemental distribution of Ca, P, and Mg on the surface of brackets was revealed by energy-dispersive spectroscopy elemental analysis. The results demonstrated that the weight percentages of Ca, Mg, and P on the surface of the AFF group was significantly lower than those of blank and control groups (Figure 7D-F), indicating that AFF treatment could inhibit mineral deposition on the bracket surface.

## Discussion

The surface quality of brackets is a critical determinant of clinical efficacy in fixed orthodontic treatment. However, conventional polishing techniques, like magnetic polishing, are limited by their compromised ability to access and finish intricate structures within brackets. AFF is an advanced finishing process designed for high-precision surface treatment of complex geometries, characterized by the extrusion of a semisolid abrasive medium through workpieces.^16^^,^^20^ To the best of our knowledge, this study is the first to investigate the polishing effects of AFF on orthodontic brackets. Our results demonstrated that AFF substantially outperformed conventional magnetic polishing by creating a more uniform and smoother surface, without causing significant alterations in slot dimensions. This superior surface quality further translated into decreased frictional resistance, inhibited bacterial adhesion, and reduced calculus-like mineral deposits, indicating that AFF is a promising strategy to improve the mechanical and biological performance of orthodontic brackets.

Orthodontic brackets are characterized by complex geometries with narrow dimensions and sharp edges, such as slots, which pose a challenge for conventional polishing approaches. To examine the polishing efficacy on intricate geometries, brackets with complex slot structures (YL, XIHUBIOM) were used in the present study. We showed that the conventional magnetic polishing reduced the surface roughness to some extent, but evident pits and protrusions were still present. This limitation could result from the rigid nature of the polishing medium, the magnetic steel needle, which was unable to conform to the shape and structure of slots. In contrast, the AFF utilized the semisolid viscoelastic abrasive medium that could be extruded through the complex geometries of brackets under hydraulic pressure.^16^ This property allows the abrasive medium to precisely conform to the intricate structures of slots, eliminating the irregular asperities. SEM micrographs exhibited a smoother and more regular surface in both the outer and slot areas following AFF treatment. Moreover, the multiscale evaluation highlights the superiority of AFF treatment compared to conventional magnetic polishing. 3D profilometry showed that magnetic finishing decreased Rq values at the microscale compared to the blank group, whereas AFM showed no significant differences at the nanoscale. This discrepancy suggests that although magnetic finishing reduced micro-asperities, it failed to remove nanoscale defects. By comparison, AFF treatment led to a substantial reduction in both Ra and Rq values at the micro- and nanoscale compared to the blank group, as revealed by AFM and 3D optical profilometry. Additionally, no significant changes in slot dimensions were observed following AFF treatment, ensuring the proper torque expression. This preservation results from the unique rheological properties of the viscoelastic medium, which preferentially acts on sharp asperities rather than flat slot surfaces.^17^^,^^21^ Collectively, these results suggest that AFF markedly improved the surface quality of brackets.

Excessive friction between brackets and archwires compromises the efficiency and efficacy of orthodontic treatment.^1^^,^^22^ Previous studies demonstrated that reduced surface roughness of brackets was directly correlated with minimized friction force.^2^^,^^3^^,^^23^ Building on the finding that AFF effectively improved the surface quality of brackets, we analysed the friction force between brackets and archwires in the present study. The results showed that the AFF group exhibited significantly lower kinetic friction than the control group. This decreased friction force may further promote efficient tooth movement and avoid undesirable adverse effects in orthodontic treatment, highlighting the advantages of AFF.

Beyond mechanical performance, the biological response to brackets is an important consideration in orthodontic treatment. Surface topography is a critical factor in bacterial adhesion.^24^ Existing studies have demonstrated that bacteria tend to attach preferentially to coarse surfaces.^25^^,^^26^ The irregularities and asperities on the surface could increase the contact area and protect bacteria from shear forces, thereby facilitating bacterial adhesion.^24^ In the current study, *S. aureus*, a common Gram-positive bacterium with strong ability of biofilm formation,^27^ was used to evaluate bacterial adhesion. The results showed that the rough surface in the blank group provided a favourable niche for bacterial adhesion, whereas AFF processing effectively removed surface asperities and prevented bacterial attachment. In addition to bacterial adhesion, dental calculus formation has also been revealed to be positively correlated with surface roughness.^28^ Consequently, mineral deposition on brackets was evaluated in the present study. Compared to the blank and control groups, the calculus-like mineralization on the surface of brackets was markedly inhibited in the AFF group. This inhibition may be attributed to the smooth surface, where fewer pits and defects minimize deposition sites. Collectively, these results suggest the potentially beneficial effects of the AFF processing on inhibiting bacterial colonization and mineral deposition.

Despite these promising results, there are certain limitations existing in the present study. First, only a single type of bracket was investigated, and the sample size was relatively small. Second, the friction analysis and biological evaluation (bacterial adhesion and mineral deposition assays) were performed under simplified *in vitro* conditions, which cannot fully simulate the complex intraoral environment. Additionally, the wear resistance and long-term durability after AFF processing should be investigated in future studies.

## Conclusions

Altogether, this study demonstrates that AFF is a superior post-processing technique for orthodontic brackets. AFF effectively reduced surface roughness, particularly within the intricate slot area where conventional magnetic polishing technique often falls short. This enhanced surface quality further lowered frictional resistance while inhibiting bacterial adhesion and calculus deposition, suggesting its great potential for improving the efficiency of orthodontic treatment.

## Funding

This work was supported by grants from the National Natural Science Foundation of China (grant numbers 82571061, 82501205, 82470952), the Natural Science Foundation of Guangdong Province (grant numbers 2026A1515011000, 2025A1515012537, 2023A1515030015), the International Orthodontic Foundation (IOF) Clinical Research Program (grant number IOF2024E11).

## Author contributions

Fan Yang: Conceptualization, methodology, project administration, writing – original draft. Chen Zhou: Methodology, investigation, writing – review and editing. Zichun Huang: Project administration, formal analysis, validation. Meihan Chen: Investigation, visualization. Yang Cao: Conceptualization, resources, supervision. Lili Chen: Methodology, resources, supervision. Weicai Wang: Conceptualization, project administration, resources, supervision. All authors have approved the final article.

## Conflict of interest

The authors declare that they have no known competing financial interests or personal relationships that could have appeared to influence the work reported in this article.

## Appendix Supplementary materials

Image, application 1

## Footnotes

Supplementary material associated with this article can be found in the online version at doi:10.1016/j.identj.2026.109721.
