# Supplementary material for: Effects of Abrasive Flow Finishing on the Surface Quality, Frictional Resistance, and Biological Performance of Brackets
Source: Int Dent J. 2026 Jul 3;76(5):109721. doi: 10.1016/j.identj.2026.109721 (PMC13352070; doi:10.1016/j.identj.2026.109721)
Supplement: Supplementary file 1 [file mmc1.docx]

**Supplementary Figures**

**Effects of abrasive flow finishing on the surface quality, frictional resistance and biological performance of brackets**

**Authors**

Fan Yang^a,#^, Chen Zhou^a,#^, Zichun Huang^a,#^, Meihan Chen^a^, Yang Cao^a,^*, Lili Chen ^a,^*, Weicai Wang ^a,^*

**Affiliations**

1. Hospital of Stomatology, Guanghua School of Stomatology, Guangdong Provincial Key Laboratory of Stomatology, Sun Yat-sen University, Guangzhou, Guangdong, 510055, China.

^#^ These authors contributed equally to this work.

* Corresponding authors.

E-mail: [caoyang@mail.sysu.edu.cn](mailto:caoyang@mail.sysu.edu.cn) (Y. Cao); [chenlili555@mail.sysu.edu.cn](mailto:chenlili555@mail.sysu.edu.cn) (L. Chen); [wangwc3@mail.sysu.edu.cn](mailto:wangwc3@mail.sysu.edu.cn) (W. Wang).

Telephone: 86-020-83863002 (Y. Cao); 86-020-62833700 (L. Chen); 86-020-83863002 (W. Wang).


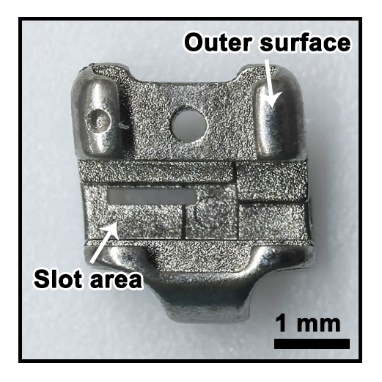


Figure S1. Illustration of regions of interest.


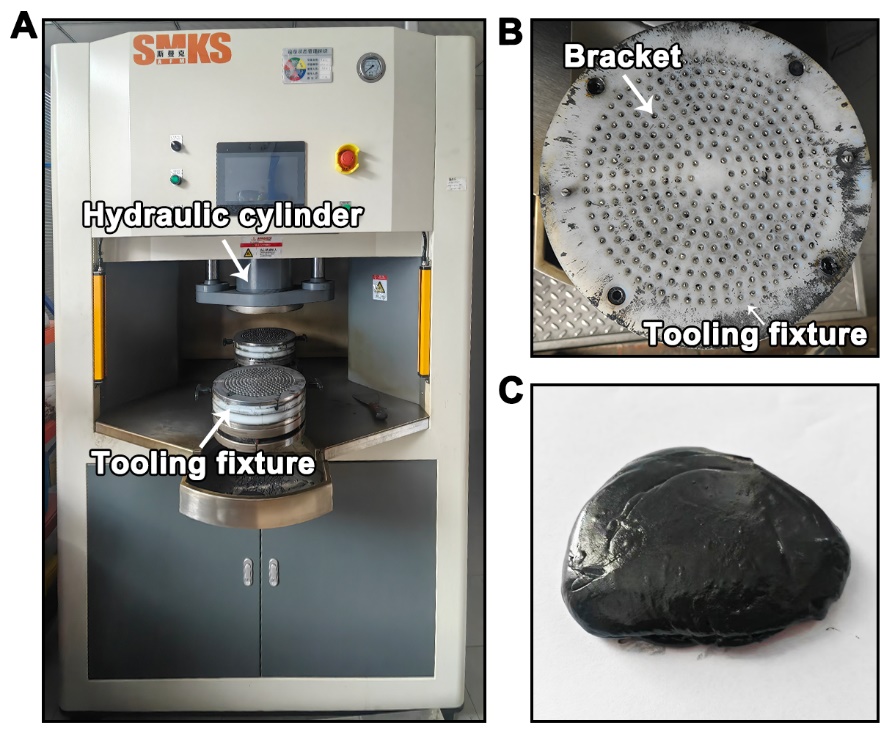


Figure S2. Abrasive flow machine. (A) Global appearance. (B) Customized tooling fixture for brackets. (C) Abrasive media.


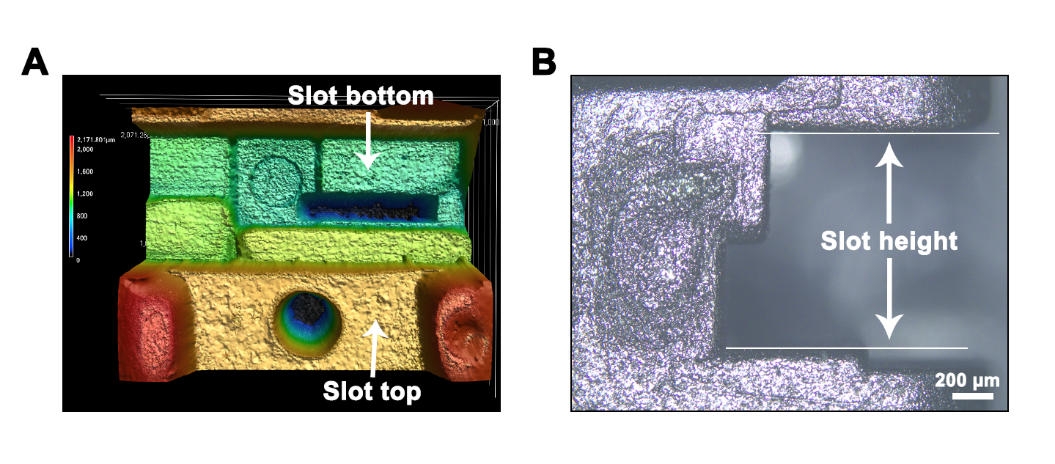


Figure S3. Illustration of slot dimensions evaluation. (A) Illustration of slot depth evaluation. (B) Illustration of slot height evaluation.
